# Case Report: A novel SERPING1 variant in a four-generation Chinese pedigree with hereditary angioedema with C1-inhibitor deficiency type 1

**DOI:** 10.3389/falgy.2026.1961416

**Published:** 2026-10-30

**Authors:** Peiyue Zhu, Yixing Luo, Shuai Dai, Cheng Jiang, Weibing Zhang

**Affiliations:** 1 Second Clinical Hospital of Wuhan University, Wuhan, China; 2 Hubei Provincial Clinical Research Center for Emergency and Resuscitation, Emergency Center, Zhongnan Hospital of Wuhan University, Wuhan, Hubei, China; 3 Department of Urology, Zhongnan Hospital, Wuhan University, Wuhan, Hubei, China

**Keywords:** C1 inhibitor (C1-INH), case report, hereditary angioedema, incomplete penetrance, SERPING1 gene, whole-exome sequencing

## Abstract

**Background:**

Hereditary angioedema with C1-inhibitor deficiency (HAE-C1INH) is a rare autosomal dominant disorder caused by pathogenic variants in SERPING1. We report a four-generation Chinese pedigree with a novel SERPING1 variant.

**Methods:**

Nine family members underwent clinical evaluation, biochemical testing (complement C4, C1-INH concentration and functional activity), whole-exome sequencing (WES), and Sanger validation. Pathogenicity was classified according to ACMG/AMP guidelines with updated ClinGen SVI specifications.

**Results:**

A novel 19-bp complex indel in SERPING1 (c.204_205delinsCCGGCCAAGAACACCAACA; p.Asn69ArgfsTer16) was identified. Sanger sequencing confirmed perfect co-segregation with C1-INH deficiency across seven carriers (five symptomatic, two asymptomatic), while two unaffected relatives were wild-type. The variant was classified as pathogenic (PVS1 + PP1_Strong+PP4 + PM2_Supporting) and absent from 1,000 Genomes, gnomAD, ClinVar, and HGMD. Two asymptomatic carriers demonstrated incomplete clinical penetrance.

**Conclusion:**

This novel SERPING1 complex indel causes HAE-C1INH type 1 in this Chinese pedigree, expanding the global variant spectrum. Family-based screening identifies asymptomatic carriers who may benefit from preventive counseling.

## 1 Introduction

Hereditary angioedema (HAE) is a rare autosomal dominant disorder, with a global prevalence estimated between 0.13 and 1.6 cases per 100,000 population (1). The disease is characterized by recurrent episodes of non-inflammatory, non-pruritic swelling affecting the skin and submucosal tissues, most commonly the face, extremities, genitals, gastrointestinal tract, and upper airway. These attacks are unresponsive to conventional therapies such as antihistamines, corticosteroids, and epinephrine, reflecting their bradykinin-mediated pathophysiology (2, 3).

HAE is primarily caused by pathogenic variants in the SERPING1 gene on chromosome 11, which encodes C1 inhibitor (C1-INH) (2, 3). According to the 2024 DANCE consensus (4), HAE is classified into HAE with C1-inhibitor deficiency (HAE-C1INH) and HAE with normal C1-inhibitor (HAE-nC1INH). HAE-C1INH is further divided into type 1 (low C1-INH antigen and function, ∼85%) and type 2 (normal/elevated antigen but impaired function, ∼15%). To date, over 748 distinct SERPING1 variants have been documented, encompassing missense, frameshift, splice-site, nonsense, and large deletion variants (5).

HAE is rare, and awareness remains low, leading to frequent misdiagnosis. In China, the average diagnostic delay is 12.64 years from symptom onset (6). For rare monogenic disorders such as HAE-C1INH, large multi-generation pedigrees offer distinct advantages: they enable robust variant validation through familial co-segregation analysis and allow identification of asymptomatic carriers that reveal penetrance patterns (7, 8). Herein, we report a four-generation Chinese pedigree with HAE-C1INH type 1 caused by a novel complex indel in SERPING1, highlighting the value of family-based genetic screening.

## 2 Case description

A four-generation Chinese pedigree with HAE was recruited from Zhongnan Hospital of Wuhan University. No consanguinity was reported. The study was approved by the Institutional Review Board of Zhongnan Hospital of Wuhan University (approval number: 2023191K), and all participants provided written informed consent.

The proband (ID 16) was a 36-year-old male presenting with recurrent episodes of facial and extremity edema since adolescence. The edema was non-pitting, non-pruritic, and unresponsive to antihistamines, corticosteroids, and epinephrine. Attacks lasted 1–5 days, with facial swelling persisting the longest. Physical examination during acute episodes revealed marked asymmetric swelling of the face and extremities without urticaria. The patient had no history of allergic diseases or autoimmune disorders.

Family history revealed multiple affected members across two consecutive generations, consistent with autosomal dominant inheritance (Figure 1). A total of nine family members were enrolled. Among them, five individuals (IDs 8, 16, 17, 22, and 29) were symptomatic with recurrent cutaneous or orofacial edema, two individuals (IDs 6 and 30) were asymptomatic carriers with biochemical evidence of C1-INH deficiency, and two individuals (IDs 7 and 27) were unaffected relatives with normal complement profiles. Notably, one suspected affected family member (ID 4) had died from acute laryngeal edema and asphyxia prior to this study, underscoring the life-threatening nature of this condition.

**Figure 1:**
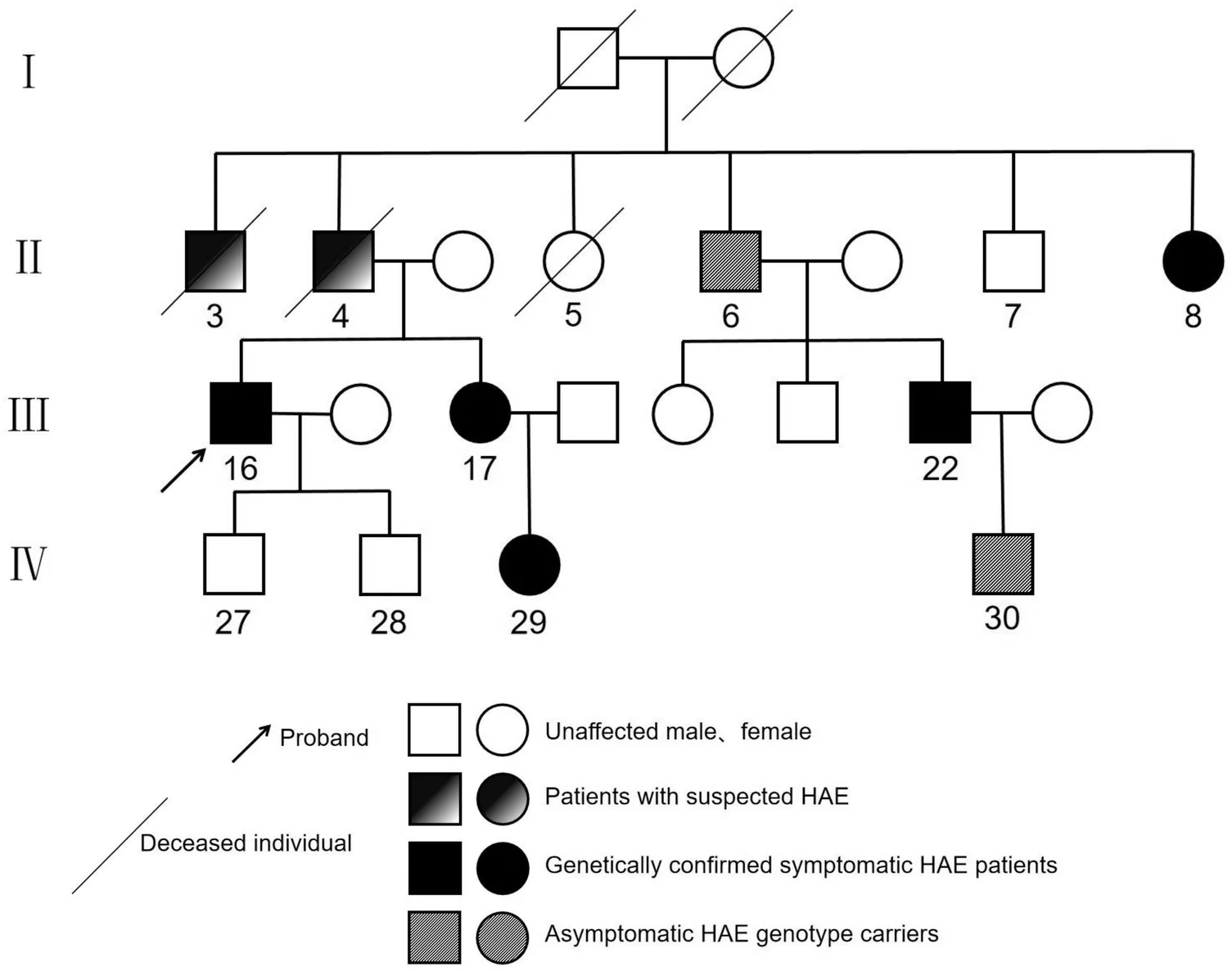
Pedigree of the proband. The proband (arrow, III-5) is a genetically confirmed symptomatic HAE patient. Filled (black) symbols=affected; open (white) = unaffected; gray=asymptomatic carriers; half-filled=suspected HAE; slashed=deceased. Squares=males; circles=females. Autosomal dominant inheritance across four generations (I–IV).

Peripheral venous blood samples (5 mL each) were collected from all nine participants. Serum C4 concentration was measured by immunoturbidimetry (reference range: 0.10–0.40 g/L), C1-INH antigenic concentration by scattered turbidimetry (reference range: 0.21–0.39 g/L), and C1-INH functional activity by ELISA (reference range: ≥68.0%; results between 41% and 67% considered borderline). All assays were performed at the Guangzhou KingMed Center for Clinical Laboratory (ISO 15189-accredited) in duplicate, with results compared against age- and sex-matched reference ranges.

Complement profiling demonstrated that seven individuals (IDs 6, 8, 16, 17, 22, 29, and 30) exhibited biochemical features of C1-INH deficiency, with markedly decreased C4, reduced C1-INH concentration, and impaired functional activity. In contrast, two family members (IDs 7 and 27) showed normal values for all three parameters. The clinical and laboratory characteristics of all nine enrolled members are summarized in Table 1, and representative photographs of angioedema attacks are shown in Figure 2.

**Table 1:** Clinical and laboratory characteristics of the nine enrolled family members in the HAE-C1INH pedigree.

Subject	Age (yr)	Age at onset (yr)	Sex	Clinical/genetic status	Complement C4		C1-INH antigenic level（g/L）		C1-INH functional activity（%）	
					Result	Clinical implication	Result	Clinical implication	Result	Clinical implication
ID 6	62	/	male	Asymptomatic carriers	0.01 g/L↓	↓	0.05	↓	5	↓
ID 7	59	/	male	Unaffected individuals	0.26 g/L	Normal	0.25	Normal	＞93	Normal
ID 8	53	Laryngeal edema at 25	female	Symptomatic HAE patients	＜0.01 g/L↓	↓	＜0.02	↓	5.6	↓
ID 16 (Proband)	36	Laryngeal edema at 23	male	Symptomatic HAE patients	0.01 g/L↓	↓	0.05	↓	4.8	↓
ID 17	40	Facial edema at 27	female	Symptomatic HAE patients	0.01 g/L↓	↓	0.04	↓	4.9	↓
ID 22	34	Abdominal pain and distension at 31	male	Symptomatic HAE patients	0.06 g/L↓	↓	0.07	↓	22.8	↓
ID 27	8	/	male	Unaffected individuals	0.24 g/L	Normal	0.27	Normal	＞93	Normal
ID 29	14	Recurrent abdominal pain at 11	female	Symptomatic HAE patients	0.02 g/L↓	↓	0.04	↓	5.6	↓
ID 30	10	/	male	Asymptomatic carriers	0.13 g/L↓	↓	0.07	↓	51.6	↓

Reference ranges: C4, 0.10–0.40 g/L (immunoturbidimetry); C1-INH concentration, 0.21–0.39 g/L (scattered turbidimetry); C1-INH functional activity, ≥68.0% (ELISA). ↓ indicates below the reference range. *ID 30 C1-INH function (51.6%) is in the borderline abnormal range (41–67%). "—" indicates data not available or not applicable. Please fill in specific numerical values and sex/age details from the original laboratory records.

**Figure 2:**
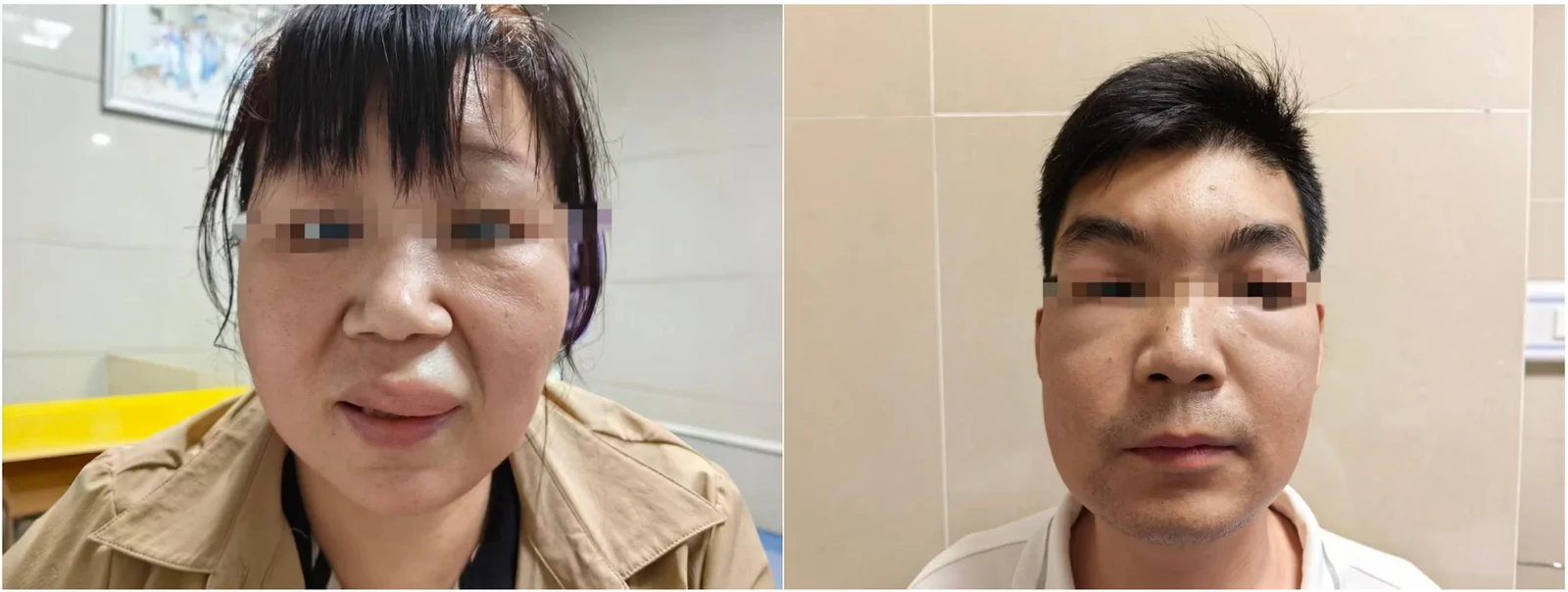
Clinical images of acute angioedema episodes. Written informed consent was obtained from all individuals for the publication of these images.

## 3 Diagnostic assessment

The diagnosis of HAE-C1INH was established according to the Chinese expert consensus criteria (9), based on recurrent non-pitting edema without urticaria, no response to anti-allergy therapy, positive family history, and biochemical evidence of reduced C1-INH concentration and/or functional activity with decreased complement C4. C1-INH functional activity below 68.0% was considered abnormal. Patients with decreased C1-INH concentration and functional activity were classified as HAE-C1INH type 1.

Genomic DNA was extracted from peripheral blood leukocytes using the QIAamp DNA Blood Mini Kit (Qiagen). Whole-exome sequencing (WES) was performed at BGI (Wuhan Branch) using the MGIEasy Universal DNA Library Prep Kit and MGI EXome Plus v1.1/v2 capture kit on the DNBSEQ-T7 platform (paired-end 100-bp reads). Bioinformatics analysis involved quality filtering (Trimmomatic v0.39), alignment to GRCh37/hg19 (BWA-MEM v0.7.17), duplicate removal (Picard v2.27.1), variant calling (GATK HaplotypeCaller v4.2.6.1), and annotation (ANNOVAR v2021-08-19) with population, clinical, and in silico prediction databases.

WES identified a heterozygous complex indel in NM_000062.3 (SERPING1): c.204_205delinsCCGGCCAAGAACACCAACA (p.Asn69ArgfsTer16). This variant results in a frameshift at codon 69, followed by 16 aberrant amino acids and a premature termination codon at codon 85, producing a severely truncated protein lacking the C-terminal serpin domain. Sanger sequencing was performed using primers E2-F (5′-TTGGGGAAAACAAAACAGAGG-3′) and E2-R (5′-GGAGGAGTAGGCTGAGAAAAGTG-3′) with annealing at 60 °C, and bidirectionally sequenced on an ABI 3730xl analyzer. Sanger sequencing confirmed perfect co-segregation: all seven carriers exhibited C1-INH deficiency, while the two unaffected relatives were wild-type (Figure 3).

**Figure 3:**
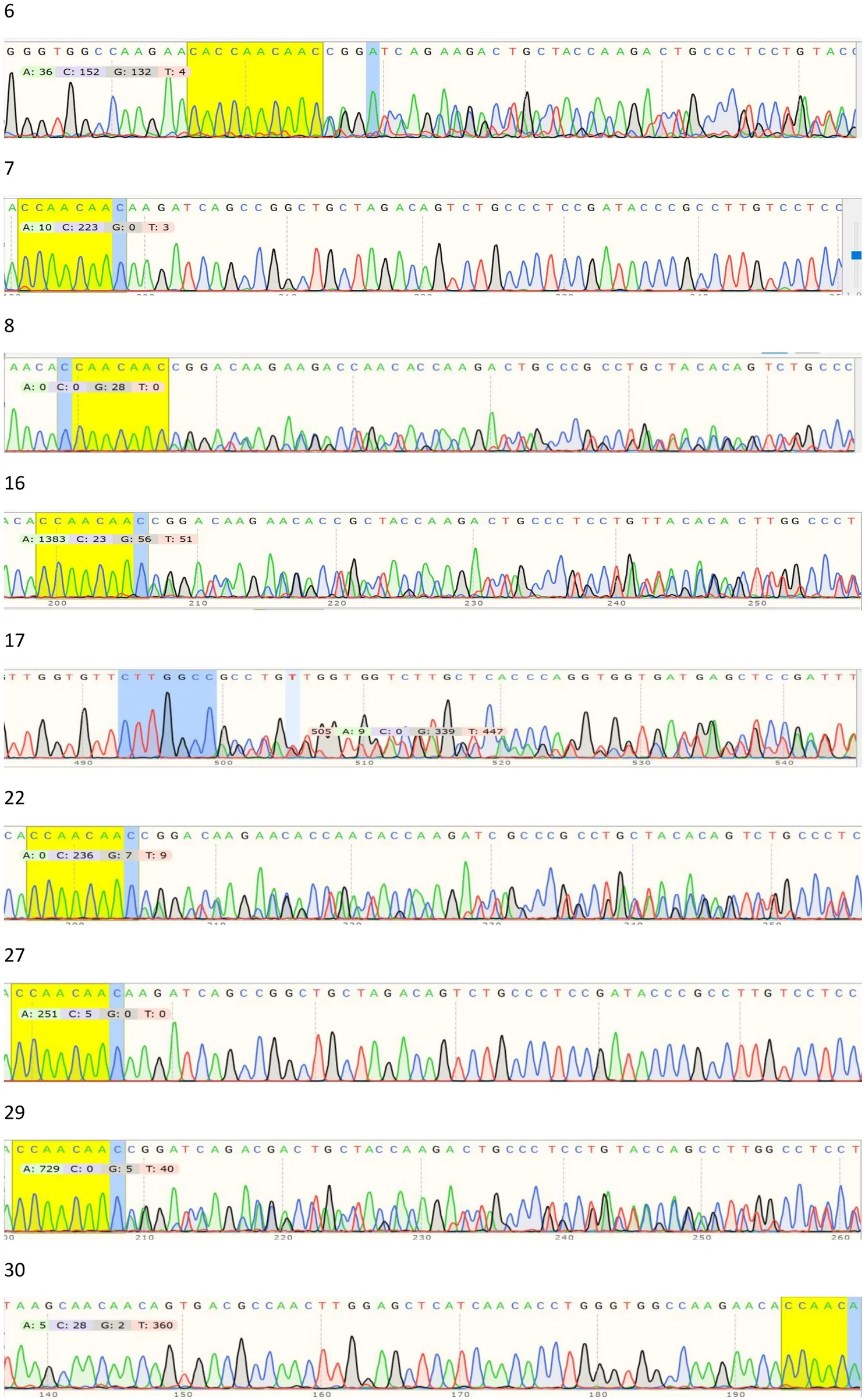
Sanger sequencing validation of the familial SERPING1 variant. The heterozygous 19-bp complex indel (c.204_205delinsCCGGCCAAGAACACCAACA, p.Asn69ArgfsTer16) was detected in all seven carriers with biochemical C1-INH deficiency but absent in the two unaffected relatives, demonstrating perfect co-segregation.

Pathogenicity was classified according to ACMG/AMP guidelines with updated ClinGen SVI specifications (10, 11): PVS1 (Very Strong: frameshift with premature termination), PP1_Strong (Strong: co-segregation across seven affected members), PP4 (Supporting: highly specific phenotype), and PM2_Supporting (Supporting: absent from control databases; PM2 downgraded per ClinGen SVI 2020 recommendation). The combination of one very strong, one strong, and two supporting criteria satisfies the threshold for “pathogenic” classification. The variant was absent from 1,000 Genomes, gnomAD, ClinVar, and HGMD, confirming its novelty.

## 4 Discussion

In this case report, we identified a novel 19-bp complex indel in SERPING1 (c.204_205delinsCCGGCCAAGAACACCAACA; p.Asn69ArgfsTer16) as the pathogenic cause of HAE-C1INH type 1 in a four-generation Chinese pedigree. This variant is located in exon 2 and has not been previously reported in any population worldwide, as it is absent from the 1,000 Genomes Project, gnomAD, ClinVar, and HGMD. Among over 748 documented SERPING1 variants (5), frameshift variants represent the second most common type (∼35%); however, complex insertion-deletion (delins) events involving simultaneous deletion and insertion of multiple nucleotides are comparatively rare. Furthermore, exon 2 is not a recognized mutational hotspot—the highest densities of type 1-associated variants are reported in exons 3 and 8 (12). The identification of this complex delins in a non-hotspot region therefore adds a structurally distinct variant to the global SERPING1 allelic series.

The clinical significance of this variant is underscored by its perfect co-segregation with the biochemical phenotype of C1-INH deficiency across the pedigree and its absence from major population databases. The predicted consequence—a severely truncated C1-INH protein lacking the C-terminal serpin domain—is mechanistically consistent with the quantitative deficiency observed in affected individuals, aligning with the established paradigm that null or loss-of-function SERPING1 variants typically give rise to HAE-C1INH type 1 (5, 13).

A particularly notable finding is the presence of two asymptomatic variant carriers (IDs 6 and 30) within this pedigree, both exhibiting biochemical C1-INH deficiency but remaining clinically unaffected throughout follow-up. This highlights the phenomenon of incomplete clinical penetrance, a well-recognized but incompletely understood feature of SERPING1-related HAE. Asymptomatic carriers have been estimated to account for approximately 5% of individuals with SERPING1 pathogenic variants in population-based studies (14). The mechanisms underlying incomplete penetrance remain elusive (15) but may involve additional genetic modifiers, environmental factors, or stochastic fluctuations in bradykinin regulation. A recent population-scale analysis suggested that SERPING1 loss-of-function variants may manifest as a polyphenotypic disorder rather than exclusively as recurrent angioedema (16). The presence of asymptomatic carriers underscores the critical value of family-based genetic screening: biochemical or genetic testing of at-risk relatives can identify individuals who may develop symptoms later in life or benefit from prophylactic counseling before high-risk situations such as surgery or dental procedures.

From a clinical perspective, this case illustrates the challenges of HAE diagnosis in China, where limited clinician awareness contributes to high rates of misdiagnosis and substantial diagnostic delays (6). The family history of a death from laryngeal edema further emphasizes the life-threatening potential of untreated HAE. HAE is mediated by bradykinin rather than histamine, rendering conventional anti-allergic drugs ineffective (3). The 2024 Chinese Expert Consensus recommends icatibant as first-line acute therapy, with lanadelumab for long-term prophylaxis (9, 17).

Several limitations should be acknowledged. First, the investigation is restricted to a single pedigree, which precludes assessment of phenotypic variability across different genetic backgrounds. Second, functional validation of the variant (e.g., *in vitro* expression studies) was not performed; pathogenicity relies on in silico predictions and co-segregation evidence. Third, the retrospective design may be subject to recall bias. Despite these limitations, the perfect biochemical co-segregation, absence from all major population databases, and clear loss-of-function mechanism collectively support the pathogenicity of this variant.

The key take-away lessons from this case are: (1) HAE should be suspected in patients with recurrent non-pitting edema unresponsive to anti-allergy therapy, especially with a positive family history; (2) biochemical testing (C4, C1-INH concentration and function) is essential for diagnosis; (3) family-based genetic screening can identify asymptomatic carriers who may benefit from preventive counseling; and (4) novel SERPING1 variants continue to expand the mutational spectrum, particularly in understudied populations.

## 5 Patient perspective

The proband reported that recurrent episodes of facial and extremity edema had significantly impacted his quality of life, leading to frequent school and work absences during childhood and early adulthood. He described multiple misdiagnoses as “allergic reactions” before the correct diagnosis was established through complement testing. Following genetic confirmation and appropriate counseling, the proband expressed relief at finally understanding the cause of his symptoms and appreciated the opportunity to have at-risk family members screened. The family reported that knowledge of the genetic diagnosis has enabled them to seek timely medical attention during acute attacks and to take preventive measures before dental procedures and surgeries. Written informed consent was obtained from the proband and all participating family members for publication of this case report.

## 6 Conclusion

In conclusion, we report a novel pathogenic 19-bp complex indel in SERPING1 (c.204_205delinsCCGGCCAAGAACACCAACA; p.Asn69ArgfsTer16) causing HAE-C1INH type 1 in a four-generation Chinese pedigree. This variant has not been reported in any population to date, expanding the global HAE variant spectrum (5, 18). The identification of two asymptomatic carriers highlights incomplete clinical penetrance and underscores the value of family-based genetic screening for early prevention and genetic counseling.

## Data Availability

The authors acknowledge that the data presented in this study must be deposited and made publicly available in an acceptable repository, prior to publication. Frontiers cannot accept a manuscript that does not adhere to our open data policies.
